# Introducing gradient severe shot peening as a novel mechanical surface treatment

**DOI:** 10.1038/s41598-021-01152-2

**Published:** 2021-11-11

**Authors:** Erfan Maleki, Sara Bagherifard, Okan Unal, Michele Bandini, Gholam Hossein Farrahi, Mario Guagliano

**Affiliations:** 1 Department of Mechanical Engineering, Politecnico di Milano, 20156 Milan, Italy; 2 Department of Mechanical Engineering, Karabuk University, 78050 Karabuk, Turkey; 3 Peen Service Srl, 40138 Bologna, Italy; 4 Materials Life Estimation and Improvement Laboratory, Sharif University of Technology, Tehran, 11365 Iran; 5 School of Mechanical Engineering, Sharif University of Technology, 11365 Tehran, Iran

**Keywords:** Mechanical engineering, Structural materials, Techniques and instrumentation

## Abstract

Shot peening is widely used for improving mechanical properties especially fatigue behavior of metallic components by inducing surface hardening, compressive residual stresses and surface grain refinement. In air blast shot peening, projection pressure and surface coverage (an index of peening duration) have been considered as major controlling process parameters; the combination of these parameters plays a critical role in the beneficial effects of shot peening. Generally in severe shot peening aimed at obtaining surface grain refinement, constant values of pressure are considered with different peening durations. Considering very high peening duration, however, the phenomenon of over shot peening, which can be identified with the formation of surface defects could occur. The present study introduces a novel shot peening treatment, here called gradient severe shot peening (GSSP) that instead of using constant projection pressure, implements gradually increasing or decreasing pressures. The gradual increase of the projection pressure acts as a pre-hardening stage for the following higher projection pressure boosting the potential of the material to tolerate the sequential impacts and thus become less prone to the formation of surface defects. The results of the experiments indicate significant fatigue life improvement obtained for GSSP treated specimens compared to the standard treatment with constant pressure. GSSP avoids the detrimental effects of over-peening, while maintaining the beneficial effects of surface nano-crystallization, surface hardening and compressive residual stresses. The notable difference in fatigue strength enhancement for GSSP treated material can be also attributed to the modulated surface morphology with lower surface roughness compared to a standard shot peening treatment with the same exposure time.

## Introduction

**Figure 1:**
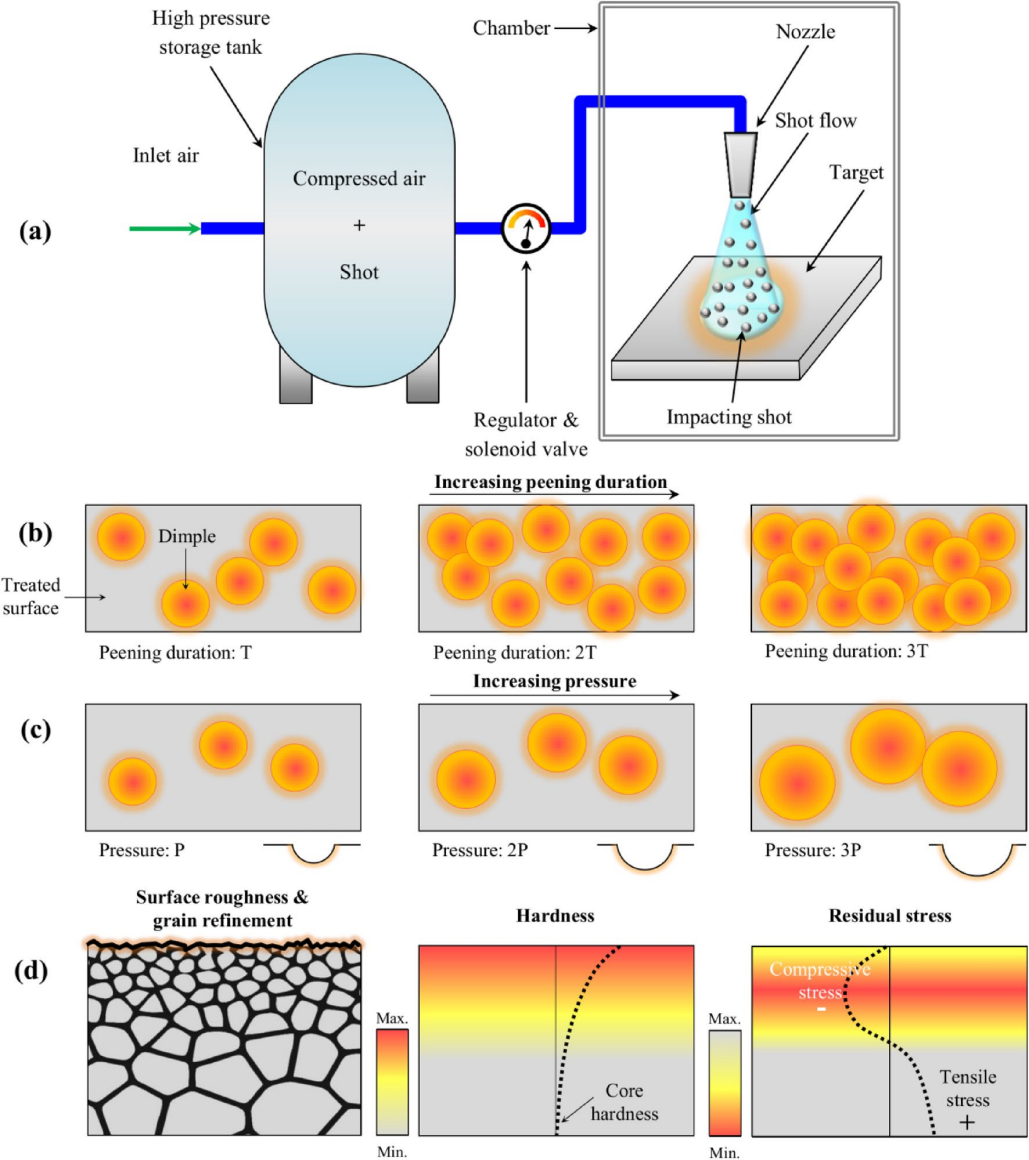
Schematic illustration of (**a**) air blast SP equipment; effects of SP treatments in terms of increasing the (**b**) peening duartion and (**c**) projection pressure on the target surface (**d**) common effects of SP treatment on increasing the surface roughness and inducing surface layer grain refinment, hardening and inducing compressive residual stresses, from left to right.

Initially presented in 1940^1^, shot peening (SP) is a cold-working process in which the surface of the target material is bombarded by impacts of small shots under controlled conditions^2^. This simple and cost-effective process is widely used for improving the mechanical properties of metallic materials such as fatigue, wear, corrosion, etc.^3–6^. Schematic illustration of an air blast SP equipment is shown in Fig. 1a. Variation of peening duration can alter the control parameter of surface coverage; coverage is defined as the ratio between the area that is plastically deformed by the impact to the total exposed area^7^. On the other hand, using a regulator and solenoid valve, the projection pressure of SP process can be controlled to regulate the velocity of the impacting shots and thus the kinetic energy of the shot stream; these are associated with another main control parameter called as Almen intensity^8,9^. Figure 1b, c schematically presents the effects of peening duration and projection pressure on the surface of the target material. The kinetic energy of the SP treatment is defined by the mass and velocity of the impacting media. In air blast SP process, high impact velocities can be obtained by increasing the projection pressure or using larger shots^5,10^. In addition, increasing the peening duration, will increase the number of the impacting shots on the target leading to higher kinetic energy transmitted to the substrate^11–15^. SP induces surface layer grain refinement and hardening as well as compressive residual stresses in the surface layer of the treated material^16^. However, due to the shot impacts and the generated dimple shaped indents, the surface morphology of the shot peened material changes leading to higher surface roughness^17,18^. Schematic illustration of surface roughness variation, grain refinement, surface layer hardening and induced compressive residual stresses are presented in Fig. 1d.

**Figure 2:**
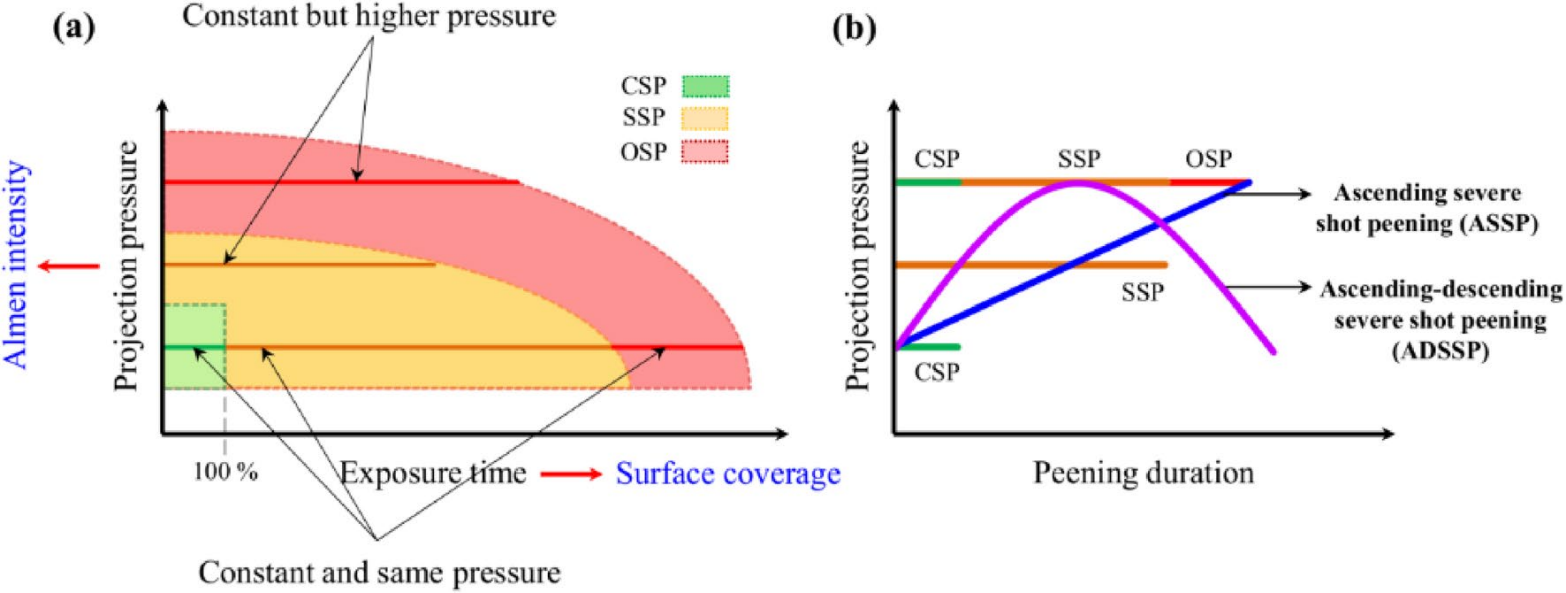
Schematic illustration representing (**a**) the comparsion of CSP, SSP and OSP in terms of projection pressure and peening duration and (**b**) variations of projection pressure through the peening duration in novel GSSP treatments of ASSP and ADSSP compared to CSP, SSP and OSP treatments.

It has been reported that by increasing the Almen intensity and surface coverage and accordingly raising the kinetic energy of the SP treatment compared to the ones used in the conventional shot peening (CSP), so called severe shot peening (SSP) or high energy shot peening (HESP) processes can be obtained^19–21^. SSP generated nano-structured grains on the surface layer^22^ and induces higher compressive residual stresses^23^. In addition, SSP was reported to have more beneficial effects compared to CSP in terms of mechanical properties and fatigue behavior improvements, when applied with optimized parameters^24–30^. However, it was found that by using higher intensities and coverages than the ones considered in SSP, over shot peening (OSP) phenomenon appears. Although, in OSP higher surface hardening and higher compressive residual stresses can be achieved but due to the very high kinetic energy of the shot impacts, multiple surface defects including nano/micro-cracks, and overlaps can form on the treated surface^31^. These defects have high detrimental effects on mechanical properties of the SP treated material, often leading to fatigue strength reduction^32–39^. Figure 2a presents the schematic illustration of different categories of CSP, SSP and OSP processes considering peening duration and projection pressure.

In this study, a novel type of SP, here called as gradient severe shot peening (GSSP) is presented for the first time, to the best of the authors’ knowledge. In GSSP, instead of using a constant projection pressure, variant pressures are considered. Herein, two different GSSP processes of ascending severe shot peening (ASSP) and ascending-descending severe shot peening (ADSSP) are introduced based on the trends considered for variation of pressure. The schematic comparisons of the newly presented treatments with standard categories are depicted in Fig. 2b. The projection pressure in ASSP is continuously ascending while in ADSSP it gradually increases to reach a maximum value and then decreases. In these two processes, the detrimental effects of OSP are avoided, while maintaining the beneficial effects of surface nano-crystallization, surface hardening and compressive residual stresses. The material considered for the investigations is Inconel 718 that is a nickel super-alloy. Inconel 718 has lots of application in different industries such as aviation and aerospace, etc. due its excellent mechanical strength and properties^40,41^. However, surface processing of Ni-based superalloys in particular with mechanical surface treatments is a challenging procedure^42–44^. Comprehensive experimental analyses in terms of microstructural characterization, roughness, microhardness and residual stresses measurements as well as axial fatigue tests are performed to compare different categories of treatments based on SP.

## Experimental procedure

### Material and specimen preparation

**Figure 3:**
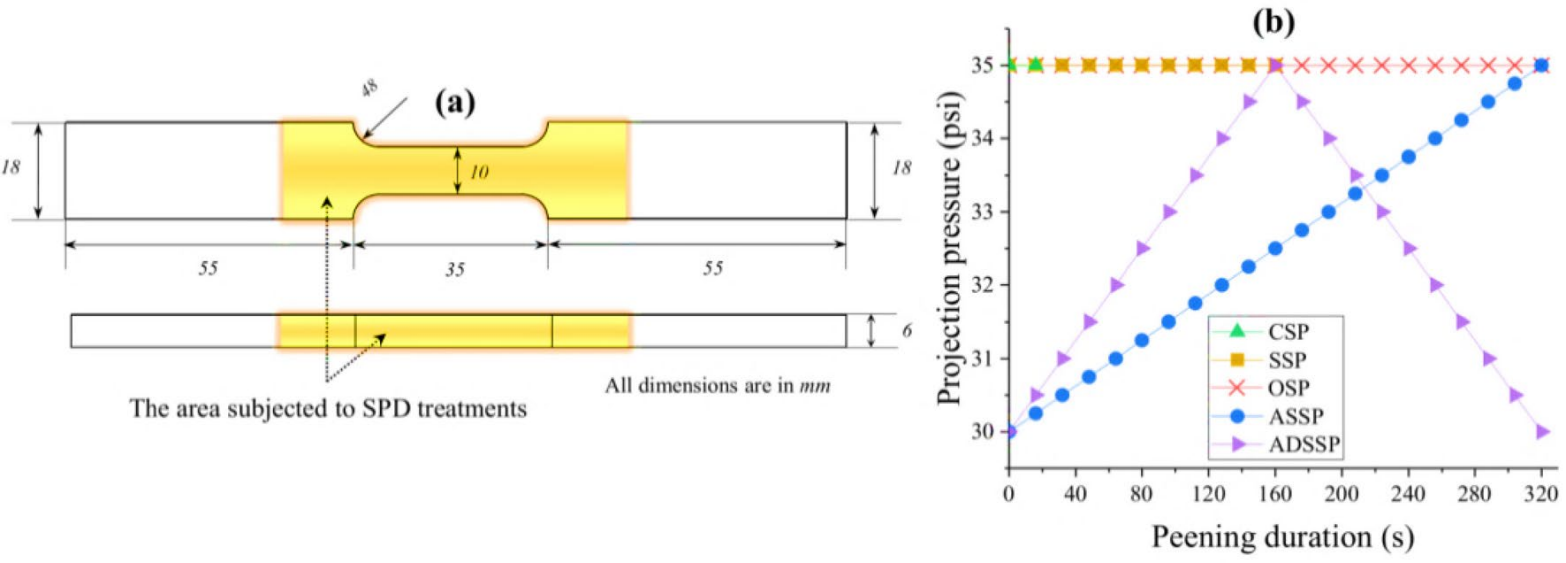
(**a**) Design of the axial fatigue test specimen and the area subjected to the SP treatments (**b**) the considered projection pressure and peening duration for the surface treatments of CSP, SSP, OSP, ASSP and ADSSP.

Cold-rolled and aged Inconel 718 nickel-based superalloy sheets with a thickness of 6 mm and the nominal chemical composition is presented Table 1, were used. The material had a yield strength of 1100 MPa, ultimate strength of 1340 MPa and elongation of 25%. Axial fatigue test specimens were prepared according to ASTM E466 standard^45^ as shown in Fig. 3a.

**Table 1:** Nominal chemical composition of Inconel 718 super-alloy (weight %).

C	Mn	Si	Cr	Co	Mo	Fe	Ti	Co	Nb	Ni
0.03	0.15	0.06	17.81	0.36	2.96	19.55	1.20	0.31	4.88	Bal

### Shot peening treatments

SP treatments were performed using air blast SP equipment. Standard S230 steel shots with a mean hardness of 52 HRC were used in all cases. A nozzle with a diameter of 6.35 mm was used for accelerating the impacting media with a stand-off distance of 10 cm. Almen intensities were obtained using standard Almen strips of type A following the procedure defined in SAE J443 standard^46^. Table 2 represents the details of the process parameters for the applied SP treatments including CSP, SSP, OSP, ASSP and ADSSP. For ASSP and ADSSP, the peening duration corresponded to that of OSP that was the longest one considered (320 s); in these cases, due to the variation of pressure, surface coverage could not be measured using the standard approach. Time intervals of 16 s (corresponding to saturation time for 15A) were considered for changing the projection pressure in GSSP treatments. Figure 3b depicts the corresponding projection pressure and peening duration for each of the considered surface treatments.

**Table 2:** Parameters of the applied SP treatments.

Specimen ID	Shot diameter (mm)	Projection pressure (psi)	Almen intensity (0.01 inch A)	Peening duration (s)	Surface coverage (%)
CSP	0.58	35	15	16*	100
SSP	0.58	35	15	160	1000
OSP	0.58	35	15	320	2000
ASSP	0.58	30 → 35	10 → 15	320	–
ADSSP	0.58	30 → 35 → 30	10 → 15 → 10	320	–

*16 s for 15A corresponds to the saturation time.

### Microstructural observations

The microstructural characterization was performed via optical microscopy using Nikon Eclipse LV150NL and field-emission scanning electron microscopy (FESEM) using high-resolution Zeiss Sigma 500 VP equipped with electron backscattered diffraction (EBSD). AZtecHKL software was used to analyze the related data. In the EBSD analyses scan area of 30 × 150 μm^2^ and 100 nm step size were considered to survey the microstructures of the specimens. The specimens were impregnated in a Phenolic hot mounting resin and ground with a series of SiC papers up to P4000. Then specimens were then polished using polycrystalline diamond water-based suspensions and etched for 8 s by a solution of 100 mL distilled water, 100 mL hydrochloric acid, 50 g ammonium hydrogen fluoride, 10 g copper (II) sulfate and 2 g potassium disulfide.

### XRD crystallite size measurements

XRD measurements were performed for obtaining the surface grain size after SP treatments of high kinetic energy including SSP, OSP and GSSP. XRD analysis was carried out using X’Pert PRO MPD (PANalytical) X-ray diffractometer and X’Pert High Score Plus (V.3) analyzer with CuKα radiation operating at 40 kV and 40 mA, scanning angles of 30°–150°, and irradiating an area of 10 mm. The full width at half maximum (FWHM) of the diffraction *θ* peaks were also determined and the crystallite sizes were achieved by means of Williamson–Hall (W–H) method^47^. In W–H method both size-induced and strain-induced broadening are considered. W–H equation can be used for calculating elastic properties such as micro-strain and crystallite size as follows^48^:

1 $$\beta \cos \theta = \frac{k\lambda }{t} + f\left( \varepsilon \right)\sin \theta$$

where *λ* is the wavelength, *β* is the corrected FWHM, *θ* is the diffraction angle, *k* is a coefficient close to unity (0.94) and *f*(*ε*) is the lattice strain function. *β* is evaluated using the measured FWHM via convoluting the Gaussian profile broadening *β*_*r*_, (the difference between the measured and the instrumental broadening) as follows:

2 $$\beta_{r}^{2} = \beta_{o}^{2} - \beta_{i}^{2}$$

### Microhardness measurements

Vickers microhardness measurements were performed from the top treated surface up to the depth of 500 µm using a Qness GmbH Q30A microhardness tester. A diamond Vickers indenter was used considering a load of 15 gf applied for 10 s. The measurements were performed along three parallel paths from shot peened surface with the same depth for each measurement through the core material; the average data of the three measurements are reported at each depth.

### Surface roughness measurements

Surface roughness was measured using a Mitutoyo contact stylus profilometer. The measurements were performed on three random locations to determine the main roughness parameters of R_a_ (arithmetic mean deviation of the assessed profile), R_z_ (maximum height of the profile) and R_t_ (total height of the profile). The roughness parameters were analyzed according to ISO 4287 standard^49^. In addition, Huvitz Digital Microscope HDS-5800 was used to analyze the surface morphology.

### Residual stress measurements

The distribution of induced residual stresses by SP treatments were measured using XRD analysis via Xstress 3000 G2/G2R X-ray Stress Analyzer (radiation Cr Kα, λKα1 = 2.2898 Å, irradiated area of 4 mm diameter, sin^2^*ψ* method, and diffraction angle (*2θ*) ~ 156° scanned between 45° and − 45°). Measurements were carried out from the top surface to the depth of 500 µm by removing a thin layer of material with a 20 µm step size using electro-chemical polishing with a solution of acetic acid (94%) and perchloric acid (6%).

### Fatigue test

Fatigue behavior of the as-received (AR) and SP treated specimens were investigated via SANTAM SAF 250kN axial fatigue test equipment with a frequency of 20 Hz. Tension-tension loading conditions with stress ratio of R = 0.1 and three constant maximum stresses of 800, 900 and 1000 MPa were considered. The fatigue tests were carried out at room temperature. Three specimens were tested from each set and the average fatigue lives were reported.

## Results and discussions

**Figure 4:**
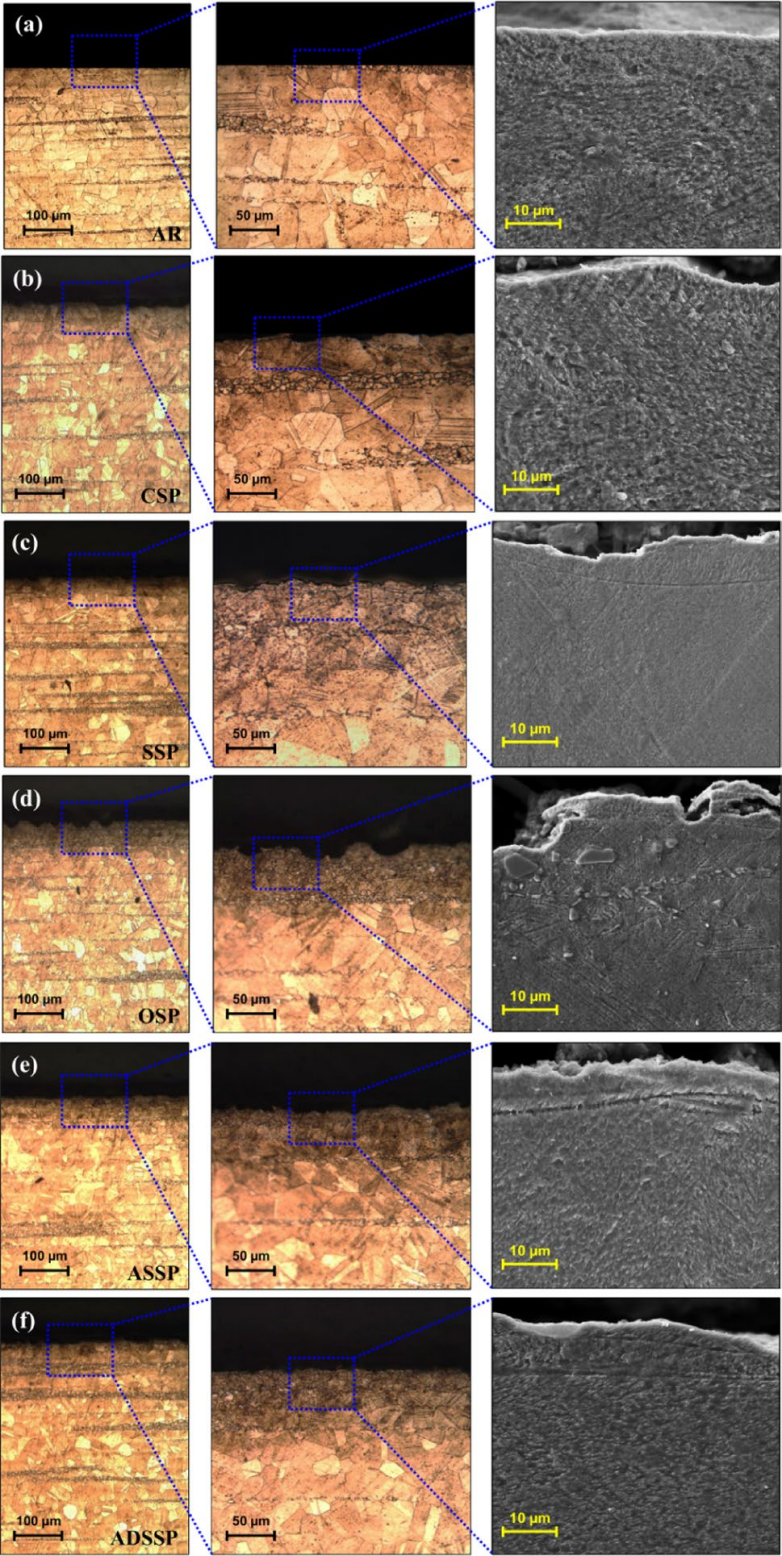
Cross-sectional OM and FESEM observations of the treated specimens of (**a**) AR core material, (**b**) CSP, (**c**) SSP, (**d**) OSP, (**e**) ASSP and (**d**) ADSSP exhibiting the plastically deformed surface layer and surface roughness.

Figure 4 reveals the cross-sectional OM and FESEM micrographs of AR and the treated specimens in different magnifications. It can be observed that by increasing the kinetic energy starting from CSP, the depth of the plastically deformed layers increased. In addition compared to the AR specimen which had flat surface (Fig. 4a), surface of the treated specimens become rough (Fig. 4b–f) due to dimple shaped deformations formed by impacting shots. For the specimens treated with high energy treatments including SSP, OSP, ASSP and ADSSP (all treated with high exposure times), the variations of surface roughness can be clearly observed in FESM cross-sectional micrographs (Fig. 4c–f). As depicted in Fig. 4d due to very high kinetic energy of OSP and unfavourable conditions of peening, multiple surface defects and irregular features can be detected on the surface. These surface defects can act as stress concentration sites leading to detrimental effects on the fatigue performance of the specimens by promoting early crack initiation. The SSP and OSP treatments differ just in their exposure times; the results indicate that after a particular peening duration at the same Almen intensity, multiple defects can be created on the surface layer potentially overshadowing the beneficial effects of the treatment.

However, in the presented GSSP treatments, which are applied with the same exposure time as OSP treatment, the gradual increase of the projection pressure can be considered as a pre-hardening stage for the following higher projection pressure; this step-by-step increase will boost the potential of the material to tolerate the sequential impacts and thus less surface defects are formed. Regarding the ASSP and ADSSP treatments, as the pressure grows ascendingly in the whole peening duration of ASSP, instead of forming dimple shaped morphologies like the ones formed in SSP (Fig. 4c), relatively smoother surface with a lower hierarchical roughness can be obtained (Fig. 4e). In addition, in ADSSP, smoother surface with small dimple shaped morphology as well as lower roughness can be achieved (Fig. 4f).

In ADSSP, the first ascending stage is mostly used for applying plastic deformation, but the second descending stage rather than its plastic deforming effects, acts like a re-peening process to decrease the surface roughness and modify the surface morphology. Re-peening treatments after initial higher energy peening has been recognized to be efficient in reducing surface toughness of metallic materials^21^. Overall, the results indicate that the GSSP treated specimens did not represent surface defects and exhibited smoother surfaces compared to the other treatments, as later confirmed by quantitative surface roughness measurements.

**Figure 5:**
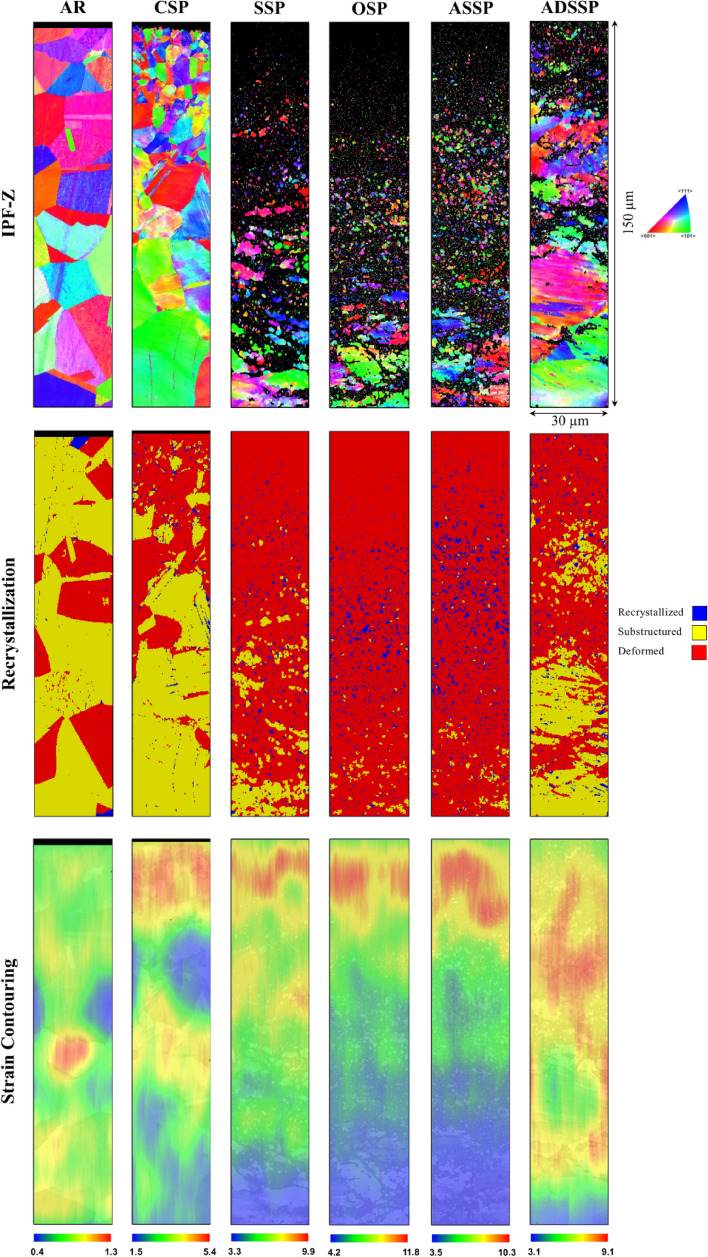
EBSD analyses in terms of IPF-Z, dynamic recrystallization and strain contouring maps for all sets of specimens.

To assess the effects of each SP treatment on surface grain refinement, three methods of EBSD, OM and XRD analyses were employed. The results of different analyses of inverse pole figures in perpendicular direction to the cross-sections (IPF-Z), recrystallization and strain contouring carried out by processing the EBSD results for the surface layer of the all sets of specimens are presneted in Fig. 5. IPF-*Z* maps reveal formation of gradient microstructures after applying SP treatements compared to the AR specimen which was not treated. The balck areas in the treated specimens with high kinetic energy including SSP, OSP, ASSP and ADSSP are reffered to the nano-structured grains whit grain size lower than 100 nm which could not detected with the considered scanning step size. Considering a rectangular area with dimensions 30 × 60 µm^2^ in the top surface of AR and CSP specimens, the mean grain area of 35.6 and 14.6 µm^2^ were obatined by AZtecHKL software for AR and CSP specimens, respectively. In addition, the results indicate that the OSP and ADSSP have the highest and lowest depth of nano-structured layer respectively.

In addition, recrystalliazation graphs which illustrate the distributions of recrystallized, substructured and deformed grains depict that amounts of plastically deformed grains are increasing after applying SP treatments. The deformed grains compared to AR specimen with 27% are increased up to 37, 84, 93, 87 and 69% for CSP, SSP, OSP, ASSP and ADSSP respectively.

Strain contouring maps can depict the plastic strain variations of material in AR and treated states. The achieved strain contouring maps reveal that the strain enhanced from the bulk material to the treated surface. The maximum strain values of the localized deformation obtained as 1.3, 5.4, 9.9, 11.8, 10.3 and 9.1 for AR, CSP, SSP, OSP, ASSP and ADSSP specimens respectively. As expected, in the treatment with high kinetic energy, due to the very high grain refinment and surface layer hardening, plastic strains are remarkably increased and OSP, ASSP, SSP and ADSSP are the most efficient processes in inducing plastic strains respectively.

**Figure 6:**
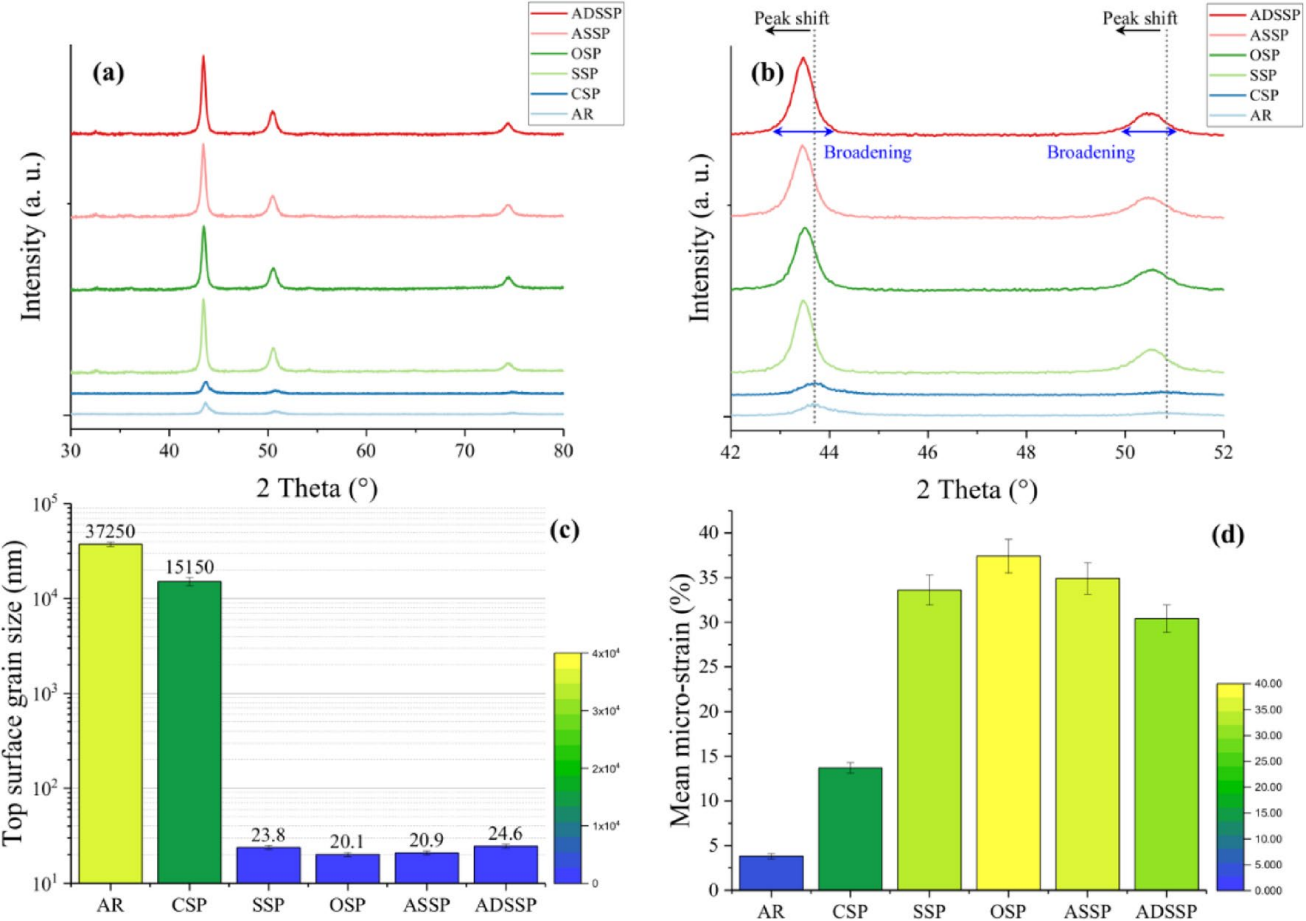
XRD patterns of the AR and SP treated specimens along different diffraction angles of 2θ in different ranges of (**a**) 30°–80°, (**b**) 42°–52° (**c**) Top surface grain size for AR and treated specimens obtained by OM for AR and CSP specimens and achieved by XRD analyses for the other severely and overly shot peened ones (**d**) Measured micro-strain in all sets of specimens obtained by XRD.

In order to obtain the grains sizes on the top surface of the specimens OM observations and XRD analyses were carried out. OM image analysis was performed on the AR and CSP series. The CSP specimen that was subjected to a lower kinetic energy process was expected to have a less notable surface plastic deformation and thus less significant grain refinement. The top surface of these specimens was gently polished removing a very thin layer of about 5 µm and subsequently etched for OM observation and image analysis. The mean grain size was measured using image analysis to be 15 µm for the CSP specimens and 37 µm for the AR one. The grain size measurement for the specimens treated with high kinetic energy was analyzed using XRD. The corresponding XRD patterns of all sets of specimens are illustrated in Fig. 6a. Figure 6b illustrates primary and secondary peaks of the XRD patterns. Compared to the AR state and CSP treated specimen which has very low plastic strain, the intensity of the primary and secondary peaks is highly increased in the treated specimens with high kinetic energy. Also, the peaks are shifted towards lower diffraction angles and peaks broadening can be observed in these sets. In the treated specimens with GSSP, the XRD pattern of the ASSP specimen showed lower intensity and wider peak compared to that of the ADSSP specimen, indicating higher extent of plastic deformation. Based on the results of OM image analyses and XRD grain size measurements, variations of surface grain size for the AR and SP treated specimens are shown in Fig. 6c. These results show a very good agreement with the obtained results by EBSD. In the AR and CSP a very good correlation is observed in terms of surface grain size. Also, the formation of nano-structured grains with size of < 100 nm (in the range of 20.1–24.6 nm) obtained by XRD confirms the formation of these grains in the black areas in the EBSD observations. The most notable grain refinement was obtained for the OSP specimen followed by ASSP, SSP and ADSSP treatments. However, it can be observed that the obtained crystallite sizes are comparable. In addition, the mean micro-strains determined in all sets of specimens as presented in Fig. 6d. As expected, OSP represented the highest micro-strain level on top surface due to the highest pressure and exposure time. However, it was found that ASSP (with a lower pressure compared to SSP but two times higher exposure time) was characterized with a higher micro-strain. ADSSP specimen exhibited the lowest micro-train level showing a slight difference with SSP. In addition, it can be observed that, the variations and trends of micro-strain values obtained by XRD are in agreement with the plastic strain variations achieved by EBSD results (see Figs. 5 and 6d).

**Figure 7:**
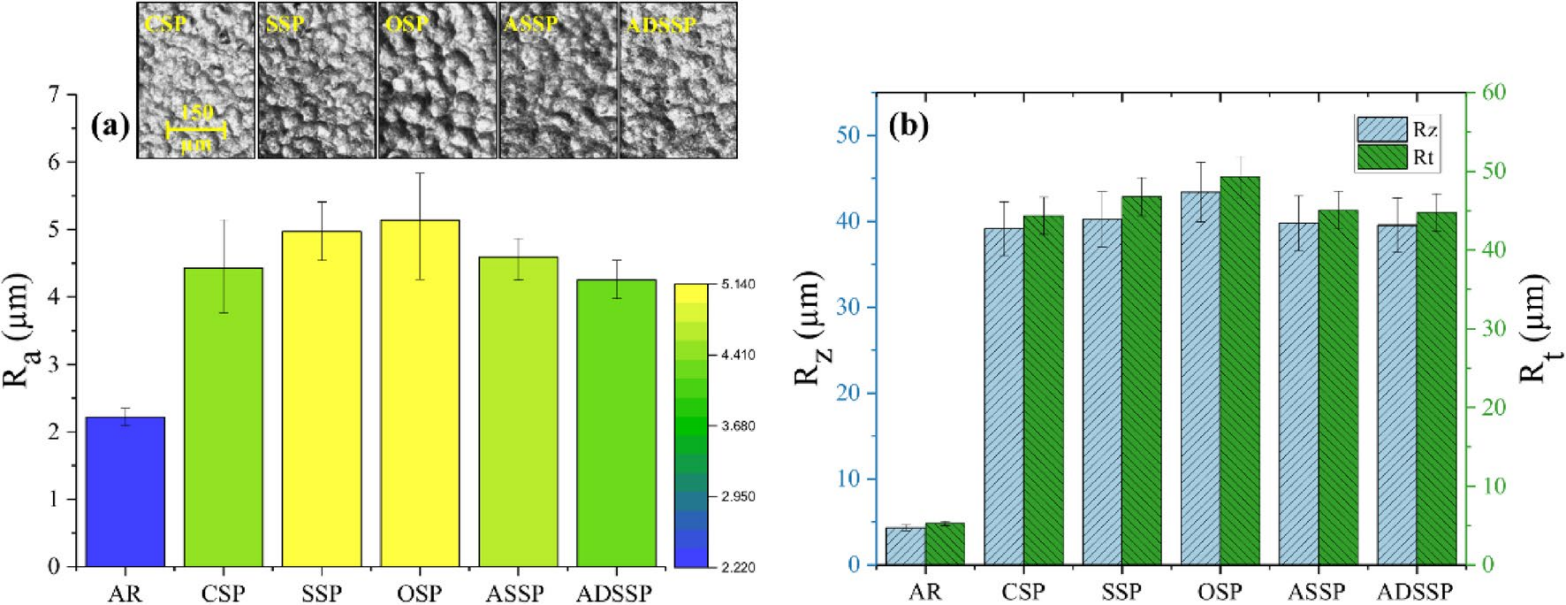
(**a**) Surface morphology of the SP treated specimens and the corresponding surface roughness in terms of R_a_ (**b**) Values of surface roughness in terms of R_z_ and R_t_.

Figure 7 depicts the measured surface roughness parameters of the AR and the SP treated specimens. All the applied SP treatments increased the surface roughness. Figure 7 reveals the surface morphology of the treated specimens and comparison of their surface roughness in terms of different standard profile parameters. The results indicate that the ADSSP specimen represented the lowest surface roughness even compared to the CSP series. Also, the surface roughness of ASSP is very close to the CSP specimen and lower than SPP and OSP. In addition, the values of standard deviations in the treated specimens with GSSP are lower than the other series, which reveals that these specimens have more uniform surface morphologies.

**Figure 8:**
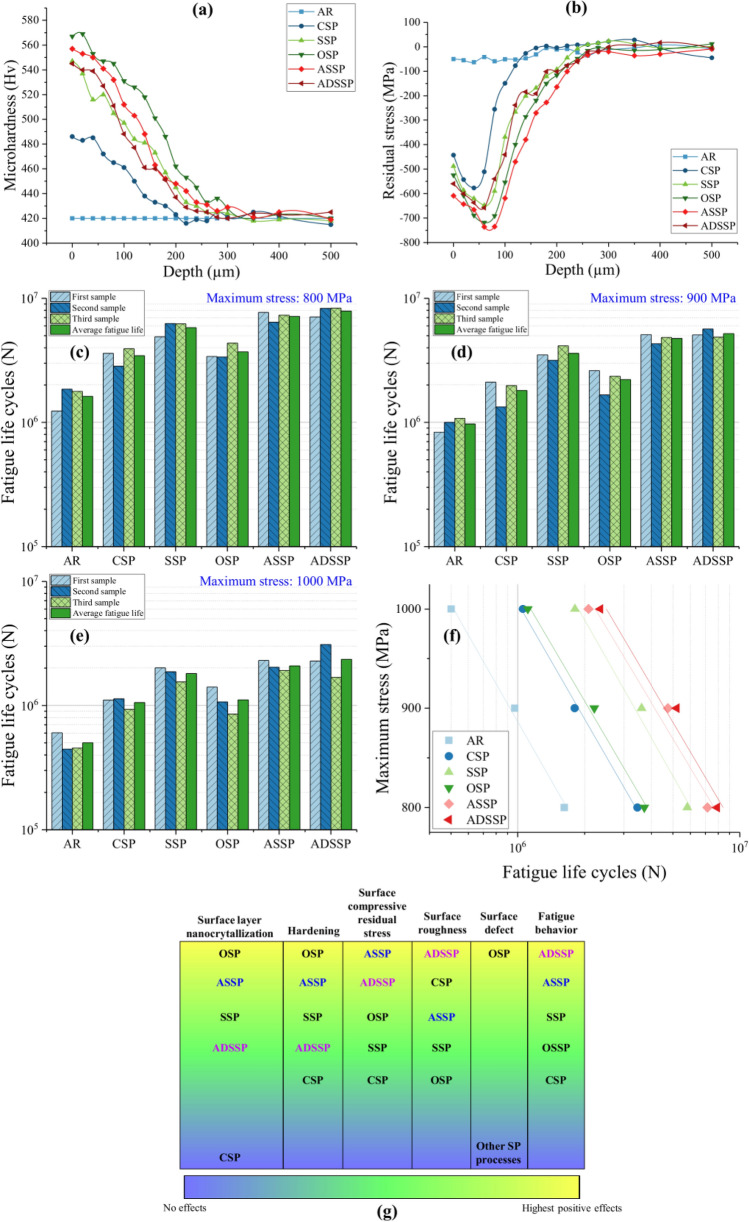
Obtained results of (**a**) microhardness profiles and (**b**) residual stresses distributions. Fatigue test results in different maximum stress levels of (**c**) 800, (**d**) 900 and (**e**) 1000 MPa. (**f**) Average fatigue life of all sets of specimens in different considered maximum stress levels. (g) Comparison of the applied SP treatments including the processes with constant projection pressure (CSP, SSP and OSP) and the ones with gradient pressure (ASSP, ADSSP).

Figure 8a shows the obtained profile of microhardness in AR and SP treated specimens from top surface up to the depth of 500 µm. OPS specimen shows the highest surface microhardness followed by ASSP. However, the surface microhardness of SSP specimen with 547 Hv is slightly higher than that of the ADSSP one, i.e., 544 Hv. The CSP treatment had the lowest effect on hardness increase due to the low kinetic energy of the peening process. Figure 8b depicts the in-depth distribution of compressive residual stresses obtained by XRD analysis. ASSP was found to be the most efficient treatments in terms of inducing compressive residual stresses on the surface and in depth. The results indicate that the gradual raise of projection pressure in GSSP treatment leads to higher compressive residual stresses on the top surface.

Axial fatigue behavior of the AR and treated specimens with different SP processes were compared at maximum stress levels of 800, 900 and 1000 MPa (Fig. 8c–e). In all considered stress levels, the results indicate that ADSSP has the highest fatigue life followed by ASSP, SSP, OSP, CSP and AR respectively. For instance the fatigue life improvement of ADSSP, ASSP, SSP, OSP and CSP specimens at maximum stress level of 900 MPa (as shown in Fig. 8d) was assessed to be about 5.3, 4.8, 3.7, 2.2 and 1.8 times higher than that of the AR specimen, respectively. As shown in Fig. 8f, similar trend can be observed in terms of obtained average fatigue life of all sets of specimens in different considered maximum stress levels. The lowest fatigue life improvement was obtained in the case of CSP specimens, despite their lower surface roughness compared to other series, mainly due to the limited grain refinement in the surface layer and lower compressive residual stresses. SSP specimens, however, exhibited a more notable fatigue life improvement compared to CSP series, due to formation of nano-structured layer on the top surface layer as well as higher compressive residual stresses, regardless their relatively higher surface roughness. In addition, the results indicated that the remarkable surface grain refinement, relatively high hardness and high compressive residual stresses, as well as lower surface roughness induced by ASSP treatment, would lead to higher fatigue lives at the same exposure time of OSP treatment, while the latter had detrimental effects on surface integrity causing reduced fatigue life. As the most important finding of this study, ADSSP specimens with nano-crystallized surface layer and the lowest surface roughness exhibited the highest fatigue life improvement despite their lower hardening compared to the other high energy SP treatments. Although ADSSP treatment caused a slightly lower hardness and compressive residual stresses compared to GSSP, it generated also lower surface roughness and a more uniform surface morphology; this combination led to an extended mean fatigue life for ADSSP treated specimens. Therefore, it can be realized that fatigue behavior of specimens treated by high kinetic energy SP is more sensitive to surface roughness rather than the hardness and compressive residual stresses. Comparison of the applied SP treatments including the processes with constant projection pressure (CSP, SSP and OSP) and the ones with gradient pressure (ASSP, ADSSP) is summarized in Fig. 8g which reveals the efficiency of the GSSP treatments in fatigue behavior improvement.

**Figure 9:**
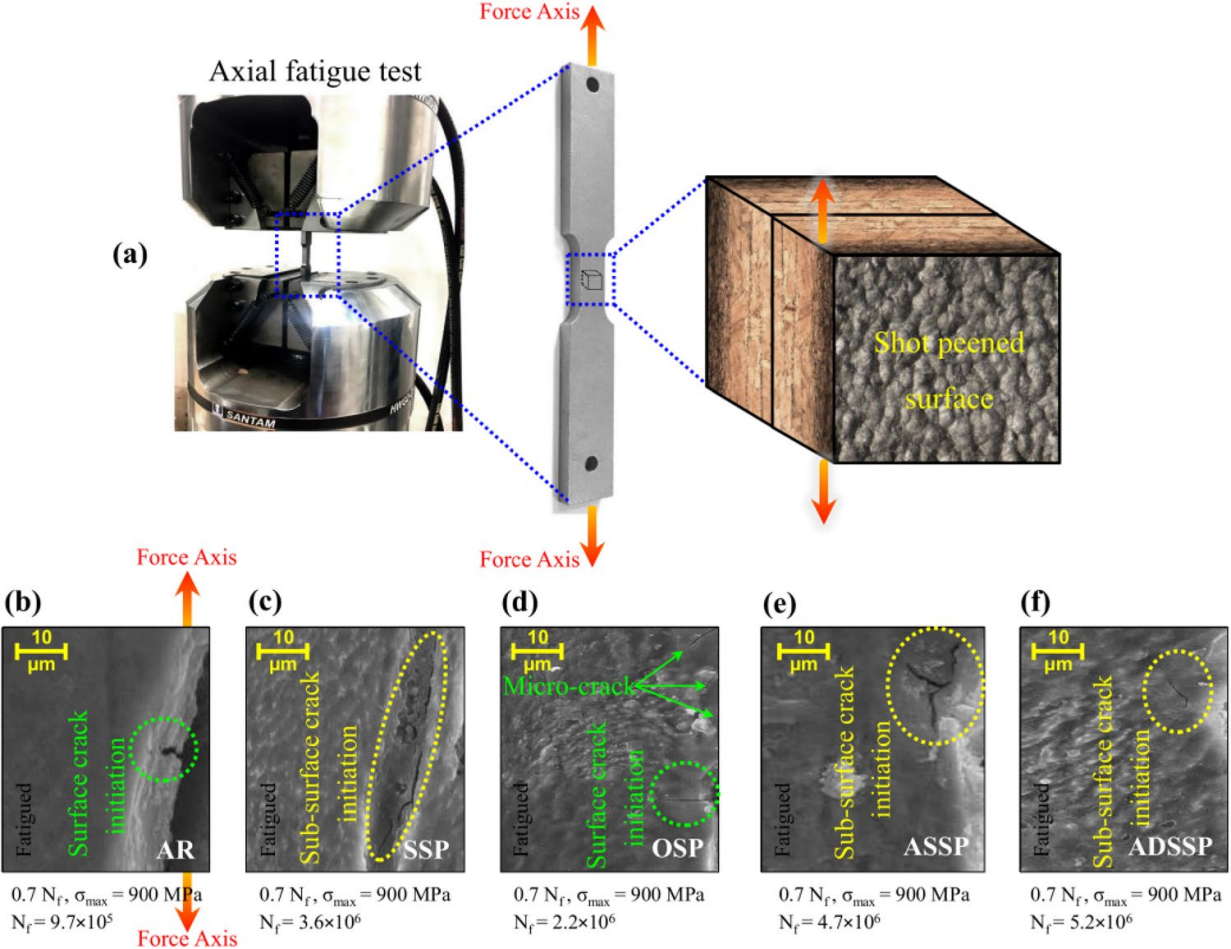
(**a**) Schematic illustration of considered scan area from analysing the crack initiations in all sets of specimens. Crack initiations sites in (**b**) AR, (**c**) SSP, (**d**) OSP, (**e**) ASSP and (**f**) ADSSP specimens.

In addition, in order to investigate the fatigue crack initiations, fatigued specimens of all sets in their 0.7 fatigue lives (0.7 N_f_) at maximum stress level of 900 MPa were analysed by FESEM observations. Firstly the specimens were cross-sectioned as shown in schematically in Fig. 9a and then surface layer of the specimens were scanned carefully to specify the crack initiations signs. In the AR specimen, the fatigue crack initiates from surface (Fig. 9b). Also, in the OSP specimen as shown in Fig. 9d, due to micro-crack formations in the top surface caused by detrimental effects of unfavourable parameters of this SP process, fatigue cracks initiate from surface through the interior. However, in the SSP, ASSP and ADSSP specimens beneficial effects of SP can be observed as the fatigue cracks are initiated from sub-surface of the treated surfaces (Fig. 9c, e, f).

## Conclusion

In air blast shot peening, the projection pressure and exposure time are considered as the major control parameters. They play a key role in defining the beneficial or detrimental effects of the shot peening treatment. Generally, constant values of pressure applied at higher exposure times are considered for shot peening treatments aimed at surface grain refinement. Based on the effcts of shot peening on the surface characteristics of the target material, three categories of shot peening treatments including conventional shot peening, severe shot peening and over shot peening can be defined. In this study, a novel class of shot peening, here called as gradient severe shot peening, is introduced for the first time. In this process, instead of using constant projection pressure, real-time variant pressures are considered. Based on the alteration trend of the projection pressure, two different processes of ascending severe shot peening and ascending-descending severe shot peening are introduced. According to the obtained results following conclusions can be drwan:

- The high projection pressure and exposure time in the gradient severe shot peening processes led to a notable surface grain refienment.
- The gradual increase of projection pressure in gradient severe shot peening acted as a pre-hardening stage for the following higher projection pressures in each step; this pre-hardening helped avoiding the formation of multiple surface defects that commonly appear at very high exposure times, as observed in the case of over shot peening.
- Under the same maximum pressure, the surface roughness of the specimens treated with gradient severe shot peening were lower than that of severely and overly shot peened specimens using a constant projection pressure. The specimens treated with ascending-descending severe shot peening showed lower surface roughness and a more uniform surface morpholgy.
- In the ascending-descending severe shot peening treatment, the second stage with descending pressure, acted as a re-peening process reducing surface roughness and further modifying surface morphology.
- Higher compressive residual stresses were obtained on the top surface by applying gradient severe shot peening due to gradual increasing of the pressure.
- Gradient severe shot peening, which resulted in surface nano-crystallization and induced relatively high hardness and compressive residual stresses, while causing a low surface roughness led to higher fatigue lives comapred to the other treatments.
- Under the same peening duration, ascending-descending severe shot peening was more effective in fatigue life enhancement compared to the ascending severe shot peening causing lower surface roughness, despite its lower hardness and compressive residual stresses. This observation indicates that the fatigue behavior of material subjected to shot peening with high kinetic energy is more sensitive to the surface roughness compared to hardness and residual stresses.
